# Structural stigma, parenthood patterns and depressive symptoms among transgender and gender diverse individuals across 30 countries in Europe

**DOI:** 10.1080/26895269.2025.2537879

**Published:** 2025-07-31

**Authors:** Felicitas Falck, B. D. Smart, L. Frisén, R. Bränström

**Affiliations:** a Department of Clinical Neuroscience, Centre for Psychiatry Research, Karolinska Institute, Stockholm, Sweden; b Department of Clinical Neuroscience, Division of Psychology, Karolinska Institute, Stockholm, Sweden

**Keywords:** legislation, population attitudes, reproduction, gender identity, mental health

## Abstract

**Introduction:**

Life circumstances of transgender and gender diverse individuals are strongly influenced by where they live. Little is known about how structural factors, for example, laws, policies, and population attitudes relating to transgender and gender diverse populations, affect their family building or mental health as parents.

**Objectives:**

We aimed to examine parenthood patterns among European transgender and gender diverse individuals, and whether their likelihood of biological parenthood differed by sex assigned at birth, gender identity or country level structural stigma. Another goal was to assess patterns of depressive symptoms by country level structural stigma based on parenthood.

**Methods:**

Using multi-level regression, we combined cross-sectional individual level data on parenthood, gender identity, sex assigned at birth and depressive symptoms from 13,302 transgender and gender diverse individuals with a measure of country level structural stigma based on national legislation, policies and population attitudes across 30 European countries.

**Results:**

In total, 8.3% were parents. Biological parenthood was more common among individuals assigned male than female. The odds ratio of biological parenthood was higher for transgender women than gender diverse individuals assigned male but lower for transgender men than gender diverse individuals assigned female. Country level structural stigma was not significantly associated with parenthood. Parenthood status and structural stigma did not predict depressive symptoms.

**Conclusion:**

Gender identity and sex assigned at birth influence family building in transgender and gender diverse individuals, most likely through societal factors and gender dysphoria. An association between structural stigma, parenthood and mental health could not be established.

## Introduction

Recent studies suggest that many transgender and gender diverse (TGD) individuals want to have children (Auer et al., 2018; Bane et al., 2024; Chen et al., 2018; Stolk et al., 2023); with proportions ranging between 54 and 82% among adults and 49–67% among adolescents (Stolk et al., 2023). However, the reported percentage of TGD individuals who become parents seems to be substantially lower at 12 and 30% (Auer et al., 2018; Bane et al., 2024; Riggs et al., 2016; Riggs & Bartholomaeus, 2018; Vyas et al., 2021). The reasons behind this discrepancy and any implications for the mental health of TGD individuals remain to be more fully explored.

Most likely due to varying sample characteristics and methodological differences across studies, the proportion of TGD individuals who wish to have children range widely. In the absence of any population-based studies, the exact proportion of TGD individuals aspiring for parenthood is unknown. Similarly, current research about the percentage of TGD individuals who are parents and what types of parenthood they have has important limitations (Carone et al., 2021); particularly outside of the US. With the exception of a US study, using a probability sample of 1,423 trans and cisgender participants, of which 19% of TGD individuals and 68% of cis-gender individuals reported being parents (Carone et al., 2021); current research on TGD parenthood is mostly based on convenience samples. Moreso, research on TGD parenthood typically relies on smaller sample sizes (Stolk et al., 2023). The limited number of larger quantitative studies describing how often TGD individuals become parents, what types of parenthood they attain and the factors that predict this leave important research gaps (Bane et al., 2024; Hahn et al., 2019; Light et al., 2018). To our knowledge no large-scale multi-country study covering these topics has been conducted in Europe, which this study aims to address.

### Stigma against TGD individuals

Unlike cis gender individuals, TGD people who aspire for parenthood do so in a setting where systemic barriers, including social stigma and discrimination (James et al., 2016), a lack of legal protections, and discrimination in health care (Falck et al., 2021; Light et al., 2014) may undermine their ability to have children as well as their health and wellbeing as parents (Carone et al., 2021). These circumstances are rooted in stigma against TGD populations. Stigma is the process whereby individuals with a common trait are identified as different, labeled and associated with undesirable characteristics and stereotypes which are used to justify their discrimination (Link & Phelan, 2001). Stigma against TGD individuals encompasses the whole of society and takes place at as well as across individual, interpersonal and structural levels, leaving a profound impact (Hatzenbuehler & Pachankis, 2016). At an interpersonal level TGD individuals are exposed to negative attitudes, discrimination and violence (Hughto et al., 2015), including in health care encounters (Kelton, 2024). Individual level stigma manifests in the thoughts and feelings that TGD people have about themselves and believe others have about them, which may limit their social interactions, openness, health (Hughto et al., 2015) and choices in life, including on family building. Structural stigma can be defined as the “societal-level conditions, cultural norms, and institutional policies that constrain the opportunities, resources, and wellbeing of the stigmatized” (Hatzenbuehler, 2016, p. 742). Structural stigma underpins laws and cultural beliefs that systematically disadvantage TGD individuals, deplete their access to resources and have a negative impact on their health, wellbeing and opportunities in life (Hatzenbuehler & Link, 2014). According the minority stress theory, initially developed by Meyer (1995) and adapted for transgender populations by Hendricks and Testa (2012), exposure to external stigma such as rejection and violence, result in anticipation of such adverse events. As a consequence TGD individuals avoid situations where stigma is expected, hide their identity and internalize negative societal attitudes toward themselves. These external realities and individual adaptations act as stressors that may have negative mental health consequences (Hendricks & Testa, 2012) and could potentially restrict important life choices such as building a family.

### Structural sigma and parenthood among TGD individuals

Structural stigma limits the reproductive health and rights of TGD individuals, according to qualitative studies (Falck et al., 2021; Saadat et al., 2024; van Amesfoort et al., 2023). Societal-level factors that are likely to influence the ability of TGD individuals to become parents include cis-gender norms regarding parenthood (Hafford-Letchfield et al., 2019); insurance regulations on fertility preservation (Chen et al., 2019); institutional barriers, normative beliefs among social workers and legal restrictions that disqualify TGD individuals from adoption or fostering (Brown, 2021, Farr & Goldberg, 2018); as well as legislation that limits their reproductive capacity (Falck et al., 2021, van Amesfoort et al., 2023). For instance, until recently most European countries mandated sterilization for anyone who wanted to change their legal gender (ILGA-Europe, 2015). In the last decade, discussions about TGD rights have led several countries to repeal such discriminatory legislation (ILGA-Europe, 2024). However, many countries still mandate sterilization or limit the life opportunities of TGD individuals through other discriminatory legislation or a lack of regulations that protect them (ILGA-Europe, 2024). Consequently, the legal situation for TGD individuals varies substantially between European countries (ILGA-Europe, 2024); allowing for cross-country comparisons in research (Bränström & Pachankis, 2021; Ünsal et. al., 2024).

### Biological, psychological and interpersonal factors affecting TGD parenthood

Aside from structural factors, a variety of biological, psychological and interpersonal aspects are likely to affect the ability of TGD individuals to become parents. Among biological factors, gender affirming hormones and surgery can reduce or permanently hamper fertility (Stolk et al., 2023). For example, testosterone often causes infertility in individuals assigned female at birth (AFAB), although this effect is usually reversible if the treatment is terminated (Mancini et al., 2020; Stolk et al., 2023). Feminizing estrogen treatment among TGD individuals assigned male at birth (AMAB) also reduces fertility (Cheng et al., 2019). While spermatogenesis may resume if the treatment is discontinued, it takes time and is not always successful (Barnard et al., 2019; Stolk et al., 2023). Gender affirming surgery, removing the testes, ovaries and/or uterus cause permanent infertility (Mancini et al., 2020). Despite these obstacles, studies indicate that most TGD parents are genetically related to their children (Auer et al., 2018; Bane et al., 2024; Riggs et al., 2016; Riggs & Bartholomaeus, 2018; Tornello et al., 2019). However, most who have undergone gender affirming treatments have become parents prior to these (Auer et al., 2018; Riggs & Bartholomaeus, 2018; Tornello et al., 2019).

Among psychological factors, gender dysphoria, that is, the distress that many TGD individuals experience when their gender identity does not correspond with their body or with how other people read their gender (Coleman et al., 2022); may act as a barrier for TGD family building (Falck et al., 2021, 2024; Stolk et al., 2023; van Amesfoort et al., 2023). Gender dysphoria may deter TGD individuals from seeking fertility preservation (Chen et al., 2019; Defreyne et al., 2020; Nahata et al., 2017) and pregnancy (Besse et al., 2020; Falck et al., 2024).

Fear of interpersonal stigma expressed through other people′s judgment may also discourage TGD individuals from having children (Falck et al., 2024; Haines et al., 2014). Interpersonal factors influencing family building include discrimination or hostility in reproductive health care (Falck et al., 2021, 2024; Stolk et al., 2023; van Amesfoort et al., 2023) and the level of family support (Chen et al., 2019; Riggs et al., 2016).

### Parenthood patterns based on sex assigned at birth and binarity

While previous studies indicate that transgender women are more likely than transgender men to be parents, (Auer et al., 2018; Riggs et al., 2016; Riggs & Bartholomaeus, 2018; Tornello et al., 2019; Wierckx et al., 2012); less is known about parenthood patterns among gender non-binary people, i.e. individuals who define their gender between or beyond the binary categories of male/masculinity or female/femininity. In a recent US study among individuals AFAB, the proportion of parents was similar among binary (13%) and gender non-binary participants (11%), but gestational pregnancy was a less common route to parenthood among transgender male parents (29%) compared to gender non-binary (38%) parents (Bane et al., 2024). It is unclear if this was due to diverging preferences or life opportunities between these groups. It remains unknown if societal level stigma affects parenthood among TGD individuals differently depending on their sex assigned at birth (SAAB) or gender identity, which warrants further attention.

### Stigma related health problems among TGD parents

On a group level, TGD people suffer from more depression, anxiety and other mental health problems compared to the general population (Bockting et al., 2013; Bouman et al., 2017). These health disparities have been linked to the negative psychological effects of stigma and discrimination that TGD individuals are disproportionally burdened by Nadal et al. (2014; Veale et al., 2017). As TGD parents do not align with cis-gender norms around parenthood, they are theoretically at increased risk of stigma-related mental health problems compared to cis-gender parents and TGD individuals who do not have children (Charter et al., 2022; Chen et al., 2018; Veldorale-Griffin, 2014). Previous smaller research studies (Falck et al., 2024; van Amesfoort et al., 2023) and scoping reviews (Greenfield & Darwin, 2021; Kirubarajan et al., 2022) stipulate that TGD individuals may be particularly vulnerable to post-partum depression due to discrimination, a lack of social support, and a disproportionate exposure to stressful life events. However, qualitative research also indicates that parenthood can strengthen TGD individuals’ self-worth, social contacts, and mental health (Charter et al., 2022). A recent study using a US probability sample, found no significant differences in mental health between TGD parents and non-parents (Carone et al., 2021). This lack of difference between parents and non-parents, was interpreted by the authors as indication that the positive aspects of having children “canceled out” the negative effects of stigma. It remains to be seen if this finding can be supported in additional studies.

### Research aims to expand the knowledge on TGD parenthood

Gaining a better knowledge of how biological, individual, interpersonal, and structural factors affect family building and mental health outcomes among TGD individuals is important as it can enhance reproductive counseling, legislation, and policy development. Also, as current research on family building among TGD individuals is largely based on interview studies in small convenience samples or research conducted in the US (Stolk et al., 2023); larger studies across a variety of contexts are warranted. The cross-country differences in laws and attitudes toward TGD individuals across Europe offer a unique opportunity to study patterns of parenthood among TGD individuals across varying structural stigma environments.

To increase knowledge in this field, we analyzed data from a large cross-European sample of TGD individuals across four aims. First, we aimed to describe the prevalence of different types of parenthood (i.e. any parenthood, biological parenthood, non-biological parenthood, and legal guardianship) among TGD individuals and to explore if parenthood varied in higher vs. lower structural stigma countries. Second, we wanted to examine if the likelihood of biological parenthood among TGD individuals differed by sex assigned at birth (i.e. female vs. male) and/or gender identity (i.e. binary vs. gender diverse identities). Third, we aimed to explore if the association between structural stigma environment and parenthood varied by sex-assigned-at-birth and/or gender identity. Fourth, we wanted to examine if depressive symptoms were equally distributed between TGD parents and non-parents, and if there was a difference in depressive symptoms among parents and non-parents living in higher vs. lower structural stigma countries.

### Hypotheses

Based on previous research, we hypothesized that biological parenthood would be the most common form of parenthood among TGD individuals and that individuals AMAB would be biological parents more often than individuals AFAB (Auer et al., 2018; Riggs et al., 2016; Riggs & Bartholomaeus, 2018; Wierckx et al., 2012). Building on qualitative studies highlighting stigma as a barrier to family building (Falck et al., 2021; Stolk et al., 2023; van Amesfoort et al., 2023); we envisioned that individuals in lower structural stigma countries would be parents more often than those living in higher structural stigma countries. In line with previous US research (Bane et al., 2024); we anticipated transgender men to be biological parents less often than gender diverse individuals AFAB. In line with previous studies, we hypothesized that depressive symptoms would be equally distributed among parents and non-parents (Carone et al., 2021); and that depression would be similar for parents and non-parents living in higher and lower structural stigma environments.

## Materials and methods

### Participants

The individual level data used in this study originates from a cross-sectional anonymous large-scale web-based questionnaire study, the European Union Lesbian, Gay, Bisexual, Transgender, and Intersex Survey, initiated by the European Union Agency for Fundamental Rights (FRA) and carried out in 2019 (European Union Agency for Fundamental Rights, 2020a). The main purpose of the study was to assess experiences of discrimination and victimization among lesbian, gay, bisexual, trans, and intersex (LGBTI) individuals living in the 28 EU member states, as well as North Macedonia and Serbia (European Union Agency for Fundamental Rights, 2020b). The current study used survey data from participants aged 18 year and older, self-identifying as transgender women, transgender men, non-binary, gender queer, gender fluid, agender or polygender. Participants with a cis-gender identity, an intersex condition, or incomplete data on any of the main variables analyzed in this study were omitted. The final dataset consisted of 13,302 TGD participants. The study protocol was approved by the Regional Ethical Review Board of Stockholm, Sweden (no. 2020-06781).

### Development of the questionnaire and recruitment

The 2019 FRA questionnaire was developed through consultation with European and EU intergovernmental bodies, researchers, and human rights organizations. It was translated into 31 languages. LGBTI volunteers validated conceptual and cognitive aspects of the survey. Informal LGBTI forums and support services facilitated the recruitment of hard-to-reach groups such as older individuals, low-income groups and those concealing their gender identity. The survey took 18 min on average to complete (European Union Agency for Fundamental Rights, 2020b).

### Country-level structural stigma index

A composite index was used as a measure of structural stigma environment toward TGD people and was calculated for each country in this study. The country-level index was developed in line with guidelines for the establishment of composite indicators (OECD, 2008; Terzi et al., 2021) and their previous use in stigma research (Bränström & Pachankis, 2021; Hatzenbuehler, 2016). Building on the theoretical conceptualization of structural stigma by Hatzenbuehler and Link (2014); legislation, policies and population attitudes were included in the index to capture both formal and informal stigma. To match the timing of the 2019 FRA survey, the index was built on population attitudes and legislation present in 2019.

#### Data on legislation and policies

We used data from the European region branch of the non-governmental organization International Lesbian, Gay, Bisexual, Transgender and Intersex Association (ILGA-Europe) concerning legislation and policies of relevance for the human rights of LGBTI people, covering all 30 countries included in this study (ILGA-Europe, 2019). ILGA-Europe had collected information on the existence or absence of national laws and policies regarding equality and nondiscrimination, family protections, hate crime, hate speech, legal gender recognition, bodily integrity, asylum and civil society conditions. By removing information only relevant for sexual minorities, we were left with regulations of importance specifically for gender minorities. In its final form the law stigma index of this study contained 36 metrics on policy/legislation, which were weighed depending on their importance for TGD people, using ILGA-Europe′s grading system.

#### Data on population attitudes

To create the composite structural stigma variable, the index on laws and policies was combined with an index on population attitudes toward TGD individuals. The attitude index relied on six questions from the 2019 Eurobarometer survey, which assessed population opinions per country, using random sampling (Kantar, 2019). In these questions participants rated how comfortable they would feel about having a transgender person as their highest political leader, a colleague at work or the partner of their child. Participants also indicated to what extent they agreed with including knowledge on transgender individuals in school curricula, the ability of transgender individuals to change their legal gender, and being able to select a neutral gender marker. The average score across these six questions was used as a population attitude index.

#### Building the composite country level structural stigma index

We merged the z-score standardized law and policy index with the z-score standardized population attitude measure to create the final composite index of country-level structural stigma. The law and policy index and the population attitude index were given equal weight and the index were coded so that a higher score indicated a greater degree of structural stigma. For two countries, Serbia and Macedonia, data on population attitudes were not assessed in the Eurobarometer and therefore their scores were only based on the laws and policies index. The correlation between the laws and policies index and the attitudes index was high (Pearson correlation 0.77, *p* < 0.001).

##### Dichotomous structural stigma country variable

We constructed a dichotomous variable to categorize countries into higher vs. lower structural stigma countries to be used in the presentation sociodemographic background information about participants. The fifteen lowest scoring countries were labeled lower structural stigma countries and the fifteen highest were labeled higher structural stigma countries.

### Self-report measures

#### Gender identity

In the FRA survey, participants were asked to report their current gender identity with the question: “How would you describe your current gender identity?” Transgender men AFAB and transgender women AMAB were categorized as gender binary. Response options for those reporting a gender queer, agender, polygender, gender-fluid, or non-binary identity were collapsed and labeled gender diverse.

#### Sex assigned at birth

Based on the FRA survey question: “What sex were you assigned at birth?” respondents were categorized as AFAB or AMAB.

#### Reproduction and being a parent

Respondents were asked to report their parenthood status using the question: “Do you have any children?” with response options: “No, I don’t have any children”; “I am a biological parent and a legal guardian of one or more children”; “I am a biological parent but not a legal guardian of one or more children”; “I am a legal guardian but not a biological parent of one or more children”; and “I raise one or more children but I am not a biological parent nor a legal guardian”. Based on their responses, participants were categorized as having or not having children. Participants were further categorized as having biological children or as having non-biological children. Similarly, respondents were categorized as legal guardians or as parents who were not legal guardians.

#### Living with children

A multiple-choice question asked participants who they lived with for “all or most of the time” with one response option being “Your or your partner′s child(ren)”. Participants selecting this option were categorized as living with children whereas those who did not were coded as not living with children.

#### Depressive symptoms

One item was used to assess depressive symptoms: “Have you been feeling downhearted or depressed over the last two weeks?” with response alternatives: “All the time”; “Most of the time”; “More than half of the time”; “Less than half of the time”; “Some of the time”; and “At no time”. Responses to the question was used to categorize respondents into those who had experienced depressive symptoms for more than half of the time during the last two weeks and those who had not.

#### Gender-affirming treatment

The FRA survey participants were asked:” Have you undergone any kind of intervention to change your body so it better matches your gender identity.” Those who indicated that they had not done so were asked if the reason for this was that they did not need it. Based on these questions we categorized participants as having received treatment or not. Those who had not accessed treatment were further categorized as having no need for treatment or as having an unmet need for treatment.

### Covariates

Survey questions assessing age, ethnicity, financial status, educational level and sex assigned at birth were used as individual level covariates in the analyses. Education differentiated between participants having a university degree and those who did not. Financial status was based on participants′ self-reported difficulties in making ends meet (i.e. “yes” vs. “no”). In sensitivity analyses, we included having received gender affirming treatment as a covariate in analyses of the link between parenthood and depressive symptoms.

At the country level, the Gini coefficient (World Bank Group, 2025) was used as a covariate. The Gini coefficient gives an estimate of income inequality. We included it as a covariate as it adjusts for potential differences between countries based on the country-level income distribution, which could potentially affect results regarding parenthood.

### Statistical analysis

Pearson′s chi-square test was used for statistical comparisons of sociodemographic differences among participants living in higher vs. lower structural stigma countries.

For our main analyses, multi-level models with a random intercept and maximum likelihood estimation were used to account for the nested data structure (i.e. individuals within countries). Individual level variables (i.e. participants’ self-reported responses to survey questions) were modeled at level 1, and the country-level structural stigma and Gini coefficient were modeled at level 2. Estimates are presented as odds ratios with corresponding 95% confidence intervals. The threshold for significance was set at *p* < .05.

To evaluate aim 1, we analyzed the prevalence of different types of parenthood in the study population (i.e. having children of any kind, biological children, non-biological children and being a legal guardian), and the association between the structural stigma index and each of these parenthood types. To answer aim 2, we analyzed the adjusted odds ratios of being a biological parent by SAAB as well as by gender identity. When individuals with a gender diverse and a gender binary identity were compared, they were separated according to SAAB. To assess aim 3, interaction effects between SAAB and structural stigma as well as between gender identity and structural stigma were included in the model predicting parenthood. To examine aim 4, we calculated the odds ratio for reporting depressive symptoms among parents and non-parents as well as the association between structural stigma and depressive symptoms. To test if the association between structural stigma and depressive symptoms varied significantly between parents and non-parents, the interaction effect between parenthood and structural stigma was included in the model.

## Results

### Descriptive statistics by country level structural stigma

Socio-demographic distribution of participants is presented by higher vs. lower structural stigma countries in Table 1. Most participants were young and AFAB, particularly in high structural stigma countries. The majority of participants did not have a binary gender identity and trans women were the least frequently reported identity.

**Table 1.** Sociodemographic and other descriptive statistics of TGD participants in the European Union Lesbian, Gay, Bisexual, transgender, and Intersex (EU-LGBTI) 2019 survey (*n* = 13,302) by higher vs lower structural stigma countries.

		Total		Higher stigma*	Lower stigma**			Chi square
		*n*	%	*n*	%	*n*	%	*p*-value
Age	18–29	9765	73.4%	2521	81.5%	7244	71.0%	<0.001
	30–39	2002	15.1%	386	12.5%	1616	15.8%	
	40–49	841	6.3%	136	4.4%	705	6.9%	
	50+	694	5.2%	51	1.6%	643	6.3%	
Sex assigned at birth	Female	8572	64.4%	2197	71.0%	6375	62.5%	<0.001
	Male	4730	35.6%	897	29.0%	3833	37.5%	
Gender identity	Trans man	3225	24.2%	765	24.7%	2460	24.1%	<0.001
	Trans woman	2588	19.5%	410	13.3%	2178	21.3%	
	Non-binary	3303	24.8%	799	25.8%	2504	24.5%	
	Gender variant	4186	31.5%	1120	36.2%	3066	30.0%	
Relationship	Live with partner	2851	21.4%	579	18.7%	2272	22.3%	<0.001
	Live separately	3136	23.6%	775	25.0%	2361	23.1%	
	No partner	7315	55.0%	1740	56.2%	5575	54.6%	
Parenthood	Live with children	653	4.9%	99	3.2%	554	5.4%	<0.001
	Have children	1100	8.3%	131	4.2%	969	9.5%	<0.001
	Legal guardian	857	6.4%	92	3.0%	765	7.5%	<0.001
	Biological children	921	6.9%	109	3.5%	812	8.0%	<0.001
Depressive symptoms	Less than half the time	6446	48.5%	1333	43.1%	5113	50.1%	<0.001
	More than half the time	6856	51.5%	1761	56.9%	5095	49.9%	
Treatment	Received	4027	30.3%	510	16.5%	3517	34.5%	<0.001
	Not needed	4287	32.2%	1234	39.9%	3053	29.9%	
	Unmet need	4988	37.5%	1350	43.6%	3638	35.6%	
Financial difficulties	Yes	6455	48.5%	1499	48.4%	4956	48.6%	0.921
	No	6847	51.5%	1595	51.6%	5252	51.4%	
Education level	No formal education	38	0.3%	9	0.3%	29	0.3%	<0.001
	Primary or lower secondary	1479	11.1%	520	16.8%	959	9.4%	
	Upper secondary	4794	36.0%	1402	45.3%	3392	33.2%	
	Post-secondary, not college or university	1804	13.6%	226	7.3%	1578	15.5%	
	University degree	5187	39.0%	937	30.3%	4250	41.6%	
Ethnic minority	Yes	990	7.4%	156	5.0%	834	8.2%	<0.001
	No	12312	92.6%	2938	95.0%	9374	91.8%	

** Higher stigma countries include Bulgaria, Croatia, Cyprus, Czech Republic, Estonia, Hungary, Italy. Latvia, Lithuania, Poland, Romania, Slovakia, Slovenia, North Macedonia, and Serbia.

* Lower stigma countries include Austria, Belgium, Denmark, Finland, France, Germany, Greece, Ireland, Luxembourg, Malta, Netherlands, Portugal, Spain, Sweden, and United Kindgom.

In total, 8.3% of participants reported having children. Most parents were biologically related to their child (83.7%) and most were legal guardians (77.9%). To have children, to live with children, to be a biological parent, or to be a legal guardian were all significantly more common among participants in lower structural stigma countries compared to higher structural stigma countries (all *p* < 0.001).

Participants living in lower structural stigma countries were less likely to report having had depressive symptoms for more than half of the time during the last two weeks and less likely to report an unmet need for gender affirming care compared to those living in higher structural stigma countries.

Although participants in lower structural stigma countries had higher education, there was no significant difference in financial status between lower and higher structural stigma countries (*p* = 0.921).

### Descriptive statistics by sex assigned at birth and gender identity

To have children, be a biological parent, and a legal guardian was twice as common among participants AMAB compared to AFAB (Table 2). The highest proportion of parents was reported among trans women (17.2%), with a much lower proportion among trans men (5.3%), non-binary (5.2%), and individuals identifying as gender queer, agender, polygender or gender-fluid (7.5%). Having received gender affirming interventions was more common among trans women (62.4%) and trans men (52.6%) compared to non-binary individuals (12.1%) and those identifying as gender queer, agender, polygender or gender-fluid (7.5%). About half of the participants reported having depressive symptoms for more than half of the time.

**Table 2.** Sociodemographic and other descriptive statistics of TGD participants in the European Union Lesbian, Gay, Bisexual, transgender, and Intersex (EU-LGBTI) 2019 survey (*n* = 13,302) by sex assigned at birth.

				Sex assigned at birth				
		Total		Female		Male		Chi-square
		*N*	*%*	*N*	*%*	*N*	*%*	*P*
Total		13,302	100.0	8572	64.4.0	4730	35.6.0	<0.001
Relationship	Live with partner	2,851	21.4	1847	21.5	1004	21.2	<0.001
	Live separately	3,136	23.6	2188	25.5	948	20.0	
	No partner	7,315	55.0	4537	52.9	2778	58.7	
Parenthood	Have children	1,100	8.3	526	6.1	574	12.1	<0.001
	Legal guardian	857	6.4	412	4.8	445	9.4	<0.001
	Biological children	921	6.9	391	4.6	530	11.2	<0.001
Treatment	Received	4,027	30.3	2176	25.4	1851	39.1	<0.001
	Not needed	4,287	32.2	2912	34.0	1375	29.1	
	Unmet need	4,988	37.5	3484	40.6	1504	31.8	
Depressive symptoms	More than half the time	6,856	51.5	4472	52.2	2384	50.4	0.051
	Less than half the time	6,446	48.5	4100	47.8	2346	49.6	

### Parenthood by structural stigma

To examine if TGD individuals living in lower structural stigma countries were more likely to be parents as compared to those living in higher structural stigma countries, we analyzed the association between country-level structural stigma and parenthood. The association between structural stigma and parenthood was not significant for any type of parenthood, i.e. for having children of any kind (Adjusted Odds Ratio [AOR] = 0.886, 95% confidence interval [CI] 0.714–1.101, *p* = 0.275), biological children (AOR = 0.971, 95% CI 0.768–1.227, *p* = 0.803), non-biological children (AOR = 0.944, 95% CI 0.822–1.084, *p* = 0.411), and being a legal guardian (AOR = 0.811, 95% CI 0.648–1.013, *p* = 0.065) (Table 3).

**Table 3.** Sociodemographic and other descriptive statistics of TGD participants in the European Union Lesbian, Gay, Bisexual, transgender, and Intersex (EU-LGBTI) 2019 survey (*n* = 13,302) by gender identity.

		Gender identity								
		Transgender women		Transgender men		Non-binary		Gender queer, Gender fluid, Agender, or Polygender		Chi-square
		*N*	*%*	*N*	*%*	*N*	*%*	*N*	*%*	*P*
Sex assigned at birth	Total	2588	100.0	3225	100.0	3303	100.0	4186	100.0	
	Female	149	5.8	3002	93.1	2469	74.8	2952	70.5	<0.001
	Male	2439	94.2	223	6.9	834	25.2	1234	29.5	
Relationship	Live with partner	605	23.4	601	18.6	704	21.3	941	22.5	<0.001
	Live separately	500	19.3	740	22.9	882	26.7	1014	24.2	
	No partner	1483	57.3	1884	58.4	1717	52.0	2231	53.3	
Parenthood	Have children	444	17.2	172	5.3	171	5.2	313	7.5	<0.001
	Legal guardian	337	13.0	125	3.9	144	4.4	251	6.0	<0.001
	Biological children	416	16.1	115	3.6	131	4.0	259	6.2	<0.001
Treatment	Received	1615	62.4	1697	52.6	401	12.1	314	7.5	<0.001
	Not needed	105	4.1	81	2.5	1555	47.1	2546	60.8	
	Unmet need	868	33.5	1447	44.9	1347	40.8	1326	31.7	
Depressive symptoms	More than half the time	1350	52.2	1674	51.9	1785	54.0	2047	48.9	<0.001
	Less than half the time	1238	47.8	1551	48.1	1518	46.0	2139	51.1	

### Biological parenthood according to sex assigned at birth

When analyzing if biological parenthood differs by SAAB, we found that individuals AMAB had a higher likelihood of having biological children than AFAB (AOR = 0.749, 95% CI 0.634–0.884, *p* < 0.001). To assess if the likelihood of biological parenthood in higher vs. lower structural stigma counties varies by SAAB, the interaction between SAAB and the country-level structural stigma was entered into the model. The interaction effect was significant (AOR = 0.742, 95% CI 0.561–0.981, *p* < 0.036), indicating that structural stigma had a different association with biological parenthood depending on what sex an individual was assigned at birth. The interaction is illustrated in Figure 1, showing a slightly larger difference in the proportion of biological parents among those living in higher vs. lower structural stigma countries for TGD individuals AFAB compared to those AMAB.

**Figure 1.**
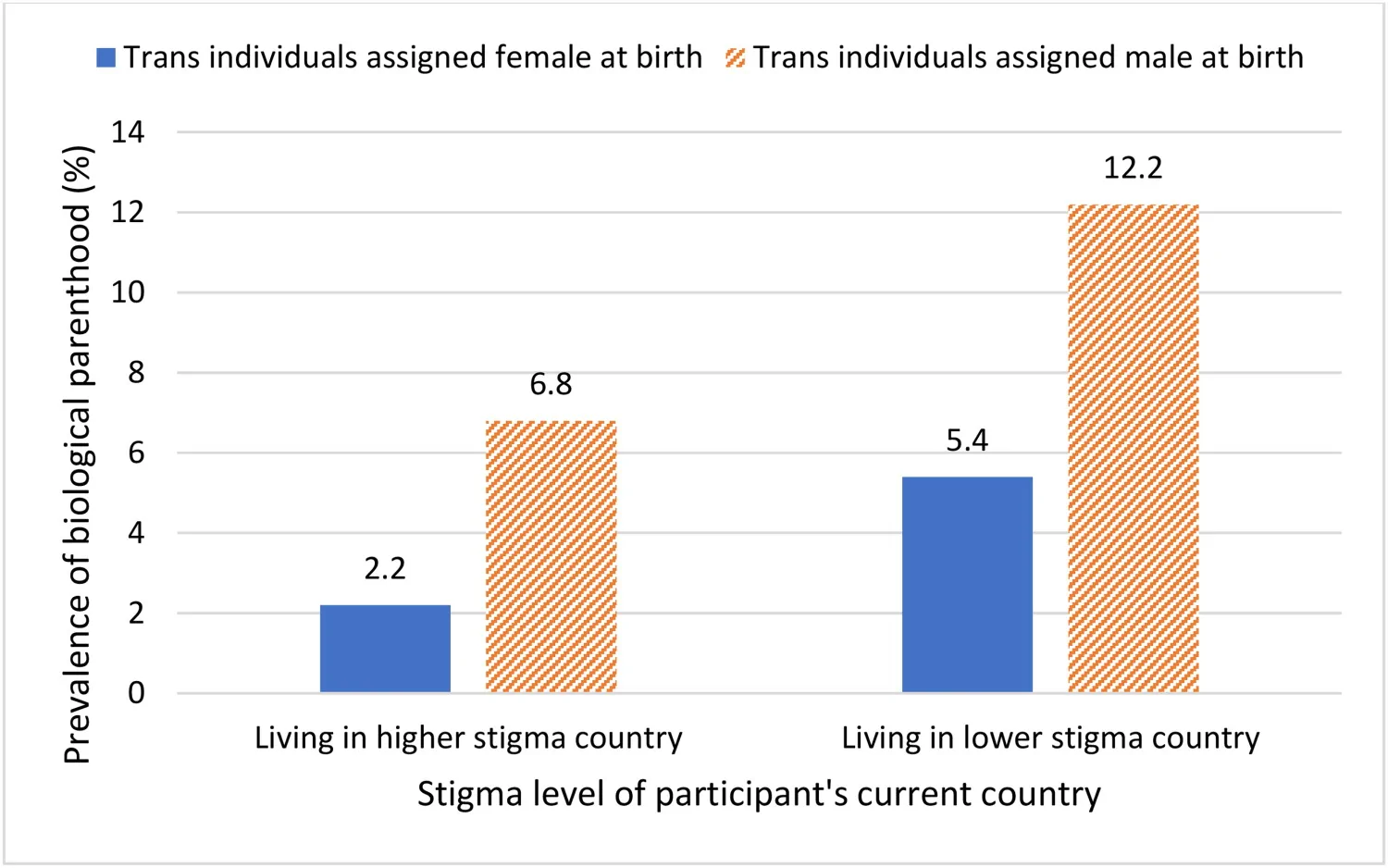
Biological parenthood prevalence among TGD participants in the European Union Lesbian, Gay, Bisexual, transgender, and Intersex (EU-LGBTI) 2019 survey (*n* = 13,302) by higher vs lower-structural stigma countries according to sex assigned at birth.

### Biological parenthood according to gender identity

Gender diverse individuals AFAB had a higher chance of having biological children than trans men AFAB (AOR = 1.362, 95% CI 1.040–1.785, *p* = 0.025). The relationship was the opposite among individuals AMAB. Gender diverse individuals who were AMAB had a lower odds ratio than trans women AMAB to have biological children (AOR = 0.563, 95% CI 0.438–0.725, *p* < 0.001). We did not find any interaction between country-level structural stigma and gender identity among individuals AFAB (AOR = 1.574, 95% CI 0.882–2.811, *p* = 0.125) nor among those AMAB (AOR = 0.760, 95% CI 0.486–1.189, *p* = 0.230).

### Depressive symptoms and parenthood

Having children was not significantly associated with depressive symptoms (AOR = 1.011, 95% CI 0.866–1.181, *p* = 0.888). Also, there was no association between country-level structural stigma and depressive symptoms (AOR = 1.021, 95% CI 0.906–1.151, *p* = 0.733) and no difference in the association between depressive symptoms and structural stigma among parents and non-parents (AOR = 1.014, 95% CI 0.787–1.306, *p* = 0.915).

### Sensitivity analyses

To test the robustness of our results we conducted several sensitivity analyses. To assess the stability of the results on depressive symptom differences between TGD parents and non-parents, we conducted a sensitivity analysis adjusting for participants′ history of having received any gender affirming interventions. After information about gender affirming interventions was entered into the model, having children remained non-significantly associated with depressive symptoms (Adj OR = 1.011, 95% 0.865–1.182, *p* = 0.888). Similarity, the association between country-level structural stigma and depressive symptoms also remained non-significant in models adjusting for information about participants’ history of gender affirming interventions (Adj OR = 1.001, 95% CI 0.887–1.128, *p* = 0.993). Lastly, a test of effect moderation with adjustment for gender affirming interventions suggested that there was no significant difference in the association between depressive symptoms and structural stigma among parents and non-parents (Adj OR = 1.017, 95% CI 0.790–1.309, *p* = 0.896).

## Discussion

By using a data set of 13,302 TGD participants living across 30 European countries with varying national structural stigma environments, we aimed to analyze parenthood patterns and their potential links to structural stigma and depressive symptoms among TGD individuals. Individuals AMAB had a higher chance of having biological children more than those AFAB. Gender diverse individuals AFAB were more likely to have biological children than trans men while trans women were biological parents more often than gender diverse individuals AMAB. Structural stigma measured at the country level was not significantly associated with parenthood or legal guardianship regardless of gender identity. There was a slightly larger difference in the proportion of individuals AFAB compared AMAB who were biological parents in higher than in lower structural stigma countries. Parenthood status and country-level structural stigma did not predict depressive symptoms.

The proportion of TGD parents in this study (8.3%) was slightly lower than what has been reported in previous research, where around 12–30% were parents (Auer et al., 2018; Bane et al., 2024; Carone et al., 2021; Riggs, 2018; Riggs et al., 2016; Vyas et al., 2021). The lower parenthood proportion in this study and its wide variation in previous studies are most likely due to study designs with studies mainly relying of data from non-probability samples. While this is one of the largest studies on TGD parenthood, population-based studies are required to determine exact parenthood patterns among TGD individuals. Such studies would also enable a comparison of parenthood prevalence between TGD populations and cisgender individuals as well as across different countries.

Contrary to our hypothesis and despite indications from some previous studies indicating structural stigma curbs the ability of TGD individuals to have children (Bane et al., 2024; Brown, 2021; Falck et al., 2021; Goldberg, 2023; van Amesfoort et al., 2023; Wesp et al., 2019); we did not find evidence for a negative association between country-level structural stigma and parenthood. The lack of support for this hypothesis can have several possible causes and structural stigma against TGD populations is likely to be associated with family building in complex ways both increasing and decreasing the likelihood of parenthood. First, structural stigma might decrease the likelihood of parenthood since stigmatizing legislation can limit TGD individuals’ access to fertility preservation, through financial, legal and institutional constraints (Bizic et al., 2018; Defreyne et al., 2020; Vyas et al., 2021); and can hamper TGD individuals’ ability to become foster or adoptive parents (Brown, 2021; Farr & Goldberg, 2018; Goldberg, 2023; Pfeffer & Jones, 2020). However, on the contrary, structural stigma might increase the likelihood of parenthood if it puts pressure on TGD individuals to live in accordance with traditional norms around gender and family values. For instance, living in countries with a more negative attitude toward TGD individuals, might increase the pressure on TGD individuals to conceal their gender identity which may include having children. Second, TGD individuals in higher structural stigma countries across Europe are less likely to access gender affirming care (Ünsal et. al., 2024). As gender affirming treatments limit fertility (Stolk et al., 2023); reducing or postponing such treatment will expand the time that TGD individuals can conceive. This could potentially increase the number of biological TGD parents in higher structural stigma countries, especially as most TGD individuals who have children do so before their social and medical transition (Auer et al., 2018; Riggs, 2018; Tornello et al., 2019). While inadequate access to gender affirming interventions prolongs the suffering from gender dysphoria (Coleman et al., 2022) and should be avoided, the timing and accessibility of gender affirming treatment could be one of several explanations for why country-level structural stigma and biological parenthood were not significantly associated in this study.

In line with our hypothesis, the majority of TGD parents in this study had biological children. This result supports previous research that highlight biological parenthood (Tornello et al., 2019; Vyas et al., 2021) and sexual activity as dominant for TGD family building (Bane et al., 2024; Haines et al., 2014; Tornello et al., 2019). Biological parenthood may be most common as other types of parenthood are more difficult to access due to legal barriers, social norms and a lack of financial resources constraining TGD individuals (Bane et al., 2024; Chen et al., 2019; Defreyne et al., 2020; Goldberg, 2023; Wesp et al., 2019).

Gender dysphoria is also a plausible explanation for results on biological parenthood of this study. Similar to other studies, TGD individuals AMAB were biological parents more often than those AFAB (Carone et al., 2021; Tornello et al., 2019). This corresponds with the higher desire for genetically related children among TGD individuals AMAB found in previous studies (Auer et al., 2018; Kyweluk et al., 2023). A probable reason for this is that the process of having a biological child puts TGD individuals AFAB at a greater risk of elevated gender dysphoria compared to the risk among TGD individuals AMAB. Pregnancy has been associated with increased reports of gender dysphoria for TGD individuals AFAB (Besse et al., 2020; Charter et al., 2018; Falck et al., 2024; Vyas et al., 2021). Concerns around body dysphoria has also been reported as a major barrier to parenthood by transgender youth (Persky et al., 2020). Consequently, gender affirming treatment often takes precedence over fertility preservation strategies such as oocyte harvesting among TGD individuals AFAB (Chen et al., 2018; Nahata et al., 2017).

Gender dysphoria may also help explain why biological parenthood was not equally distributed between individuals with gender binary and gender diverse identities in this study. Similar to previous research (Bane et al., 2024; Tornello et al., 2019); we found that trans men were biological parents less often than gender diverse individuals AFAB. Other research indicate that gender diverse individuals have less pronounced gender dysphoria than binary transgender individuals (Jones et al., 2019). This would help explain why a lower proportion of trans men than gender diverse individuals AFAB were biological parents.

Cultural norms and diverging expectations on family building for cisgender men and women are also relevant for the results of this study. Research with cisgender parents highlight that girls are socialized to prioritize parenthood more than boys (Carone et al., 2021) and express a strong desire for future biological parenthood (Quinn et al., 2013). Since cultural expectations concerning parenthood are gendered, the family building choices that TGD individuals make are likely to be affected by their sex assigned at birth as well as their gender identity. In line with this, childhood expectations of growing up to be a mother and its association with femininity has been reported to deter transgender men from undergoing pregnancy (Charter et al., 2018). While the influence of gendered socialization and cultural scripts on TGD parenthood patterns is still largely unknown (Carone et al., 2021; Chen et al., 2018); these factors could help explain the differences in biological parenthood according to sex and gender identity that were found in this study.

Gendered cultural norms concerning family building are also present in the everyday stigma that TGD parents face. TGD individuals AFAB are exposed to substantial interpersonal stigma when visibly pregnant (Falck et al., 2021; van Amesfoort et al., 2023). It appears likely that exposure to such judgmental reactions is stronger or more frequent in higher than in lower structural stigma countries. This may explain the larger difference in biological parenthood prevalence between TGD individuals AFAB compared to those AMAB found in higher as compared to lower structural stigma countries in this study.

In line with our hypothesis and a recent study that did not find any difference in mental health outcomes such as psychological distress, life satisfaction, social well-being, happiness, quality of life, alcohol and drug use between TGD parents and non-parents (Carone et al., 2021); we did not identify any association between parenthood status and depressive symptoms. As such the result of this study provides further evidence for similarities in mental health outcomes among TGD parents and non-parents. Carone and colleagues argue that the reason for their result is that children add important meaning and value to the lives of TGD parents, which might reduce or compensate for the negative effects of stigma that TGD parenthood entails (2021). Our study did not find support for such a theory, at least not on a structural level, as parenthood status did not moderate the association between country-level structural stigma and depressive symptoms. The lack of an interaction between country-level structural stigma and parenthood status for depressive symptoms, contrast to previous stigma research, which indicate that institutional barriers and interpersonal stigma in health care, social services and the everyday lives of TGD parents strain their mental health (Charter et al., 2022; Falck et al., 2024).

### Study limitations

The current study benefits from a large sample and covered a variety of countries, including eastern Europe, in contrast to previous research which has mainly focused on western countries, such as Germany (Auer et al., 2018); the US (Bane et al., 2024; Carone et al., 2021; Vyas et al., 2021) and Australia (Riggs, 2018; Riggs et al., 2016). It is the first study of TGD parenthood of this scale, include participants with a broad array of gender identities and uses a validated questionnaire. However, it also has important limitations. First, since participants were recruited online it is unknown to what extent they are representative of the TGD population in Europe. It is possible that the online recruitment and questionnaire format have resulted in a disproportionately high number of younger participants and a low proportion of parents in the sample. This limits the study’s ability to provide a valid estimate of the proportion of TGD parents. Second, power is another limitation as the number of TGD parents in some subgroups was relatively small. It is possible that smaller subgroup differences would have been significant with a large sample. As stigma is likely to prevent family building among TGD individuals in high as well as in lower stigma countries, a large data set or a very precise stigma measure are required to detect significant associations between country-level structural stigma and parenthood. Third, the survey questions had important limitations. For example, the measure of depressive symptoms was not validated and relied on one item only. A validated more extensive depression assessment tool is recommended in future studies. Likewise, the FRA survey posed very few questions regarding parenthood, limiting the analyses. The survey did not cover how participants became parents, such as through natural conception, assisted reproductive technology, surrogacy or adoption and their desire for parenthood was not explored. Also, the FRA data did not specify if participants had accessed gender affirming interventions before or after having children. Parenthood could therefore not be assessed in relation to the transition process and completion of gender affirming interventions was not included as an individual level covariate. Moreso, the parenthood prevalence measured by FRA captures the accumulation of TGD parents over several years, as opposed to the proportion who became parents in 2019. It is likely that the level of structural stigma that was present several years prior to 2019 impacted on the parenthood patterns measured in this study. Fourth, since the age of the participants′ children was unknown, the level of structural stigma the year they were born or conceived could not be calculated. Although this is a limitation, population attitudes usually evolve over an extended period (Charlesworth & Hatzenbuehler, 2025); enhancing the relevance of the 2019 CLSS index in relation to parenthood prevalence. Lastly, several structural factors that may affect parenthood among TGD individuals were unaccounted for in this study. Access to contraception, societal and religious norms, a gendered division of labor, the average living standard, and social security system all influence childbearing among cis women (Götmark & Andersson, 2020). Consequently, birth rates in the general population vary considerably between European countries, ranging between 1.81 live births per woman in Bulgaria to 1.06 in Malta in 2023 (Eurostat, 2025). It appears likely that the same factors that affect childbearing in the general population impact on TGD parenthood as well. To take national income variations into account, we adjusted for the Gini index. However, it is possible that additional country-level indicators should have been included.

### Implications for future studies

Future studies of structural stigma and parenthood would benefit from national population-based data and direct comparisons with age-matched cis-gendered controls. Longitudinal designs to capture potential shifts in parenthood prevalence and mental health outcomes over time are also encouraged. Additional studies should be conducted outside of the US. Moreso, the association between CLSS and TGD parenthood should be examined further. Such studies could focus on specific legislation and attitudes that emerge as barriers to TGD family building in qualitative studies. Moreso, studies exploring the association between stigma and mental health outcomes among TGD parents and non-parents remain few and require additional analyses.

## Conclusion

By using an unusually large data set of individual responses across Europe, this study expands the knowledge on parenthood prevalence among TGD individuals outside of the US, including in eastern Europe as well as the role of structural stigma. The findings that gender diverse individuals AFAB were more likely than transgender men to be biological parents, and that the chances of biological parenthood were greater among transgender women compared to gender diverse individuals AMAB, extend the information on gender identity as a predictor for family building. Likewise, the result that individuals AMAB were biological parents more often than those AFAB strengthens existing knowledge on differences in family building among TGD based on SAAB. The fact that parenthood status and country-level structural stigma did not predict depressive symptoms adds to the meager knowledge on mental health outcomes among TGD parents. Finally, the somewhat unexpected lack of a significant association between country-level structural stigma and parenthood, and the suggested reasons behind this result, may be used to improve the design of future studies on structural stigma and family building.

## Data Availability

The individual level data used in this study can be obtained a special license agreement with the European Union Agency for Fundamental Rights (https://fra.europa.eu/en/about-fra) upon a request.
